# Efficient 9α-hydroxy-4-androstene-3,17-dione production by engineered *Bacillus subtilis* co-expressing *Mycobacterium neoaurum* 3-ketosteroid 9α-hydroxylase and *B. subtilis* glucose 1-dehydrogenase with NADH regeneration

**DOI:** 10.1186/s40064-016-2871-4

**Published:** 2016-07-29

**Authors:** Xian Zhang, Zhiming Rao, Lele Zhang, Meijuan Xu, Taowei Yang

**Affiliations:** 1 The Key Laboratory of Industrial Biotechnology of Ministry of Education, School of Biotechnology, Jiangnan University, Wuxi, 214122 Jiangsu People’s Republic of China; 2 Jiangnan University (Rugao) Food Biotechnology Research Institute, Jiangsu Industrial Technology Research Institute, Rugao, 226500 Jiangsu People’s Republic of China

**Keywords:** 3-Ketosteroid 9α-hydroxylase, *Bacillus subtilis*, *Mycobacterium neoaurum*, 9α-Hydroxy-4-androstene-3,17-dione

## Abstract

3-Ketosteroid 9α-hydroxylase (KSH, consisting of KshA and KshB), a key enzyme in steroid metabolism, can catalyze the transformation of 4-androstene-3,17-dione (AD) to 9α-hydroxy-4-androstene-3,17-dione (9OHAD) with NADH as coenzyme. In this work, KSH from *Mycobacterium neoaurum* JC-12 was successfully cloned and overexpressed in *Bacillus subtilis* 168. The expression and purification of KSH was analyzed by SDS-PAGE and KSH activity assay. Preliminary characterization of KSH was performed using purified KshA and KshB. The results showed that KSH was very unstable, and its activity was inhibited by most metal ions, especially Zn^2+^. The whole-cells of recombinant *B. subtilis*, co-expression of KSH and glucose 1-dehydrogenase (GDH), were used as biocatalyst to convert AD to 9OHAD. The biocatalyst, in which the intracellular NADH was regenerated, efficiently catalyzed the bioconversion of AD to 9OHAD with a conversion rate of 90.4 % and productivity of 0.45 g (L h)^−1^, respectively. This work proposed a strategy for efficiently producing 9OHAD by using *B. subtilis* as a promising whole-cell biocatalyst host and co-expressing KSH and GDH to construct a NADH regeneration system.

**Electronic supplementary material:**

The online version of this article (doi:10.1186/s40064-016-2871-4) contains supplementary material, which is available to authorized users.

## Background

**Fig. 1:**
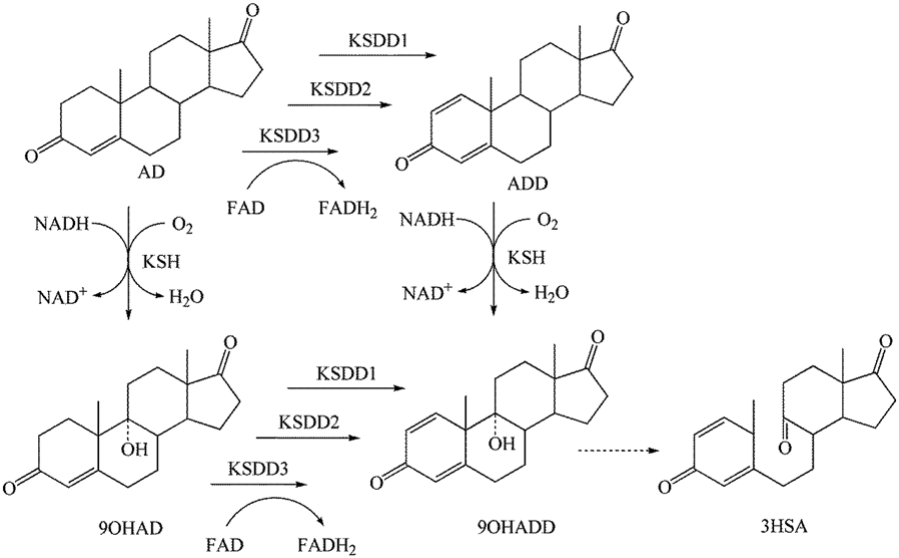
Bioconversion of AD to 9ODAD and their degradation pathway. *AD* 4-androstene-3,17-dione, *ADD* androst-1,4-diene-3,17-dione, *9OHAD* 9α-hydroxy-4-AD, *9OHADD* 9α-hydroxy-4-ADD, *KSH* 3-ketosteroid 9α-hydroxylase, *KSDD* 3-ketosteroid-∆^1^-dehydrogenase, *3HSA* 3-hydroxy-9,10-secoandrost-1,3,5(10)-triene-9,17-dione

9α-Hydroxylated steroids are important precursors in the synthesis of steroidal hormone pharmaceuticals, which have been attracted increasing attention (Donova and Egorova 2012; Donova 2007; Fernandes et al. 2003). Highly specific reactions are required to produce functionalized compounds with therapeutic use and commercial value. Due to the high region- and stereo-selectivity of the reactions, together with the mild conditions required, the high yield biological production process, which are more environmentally friendly than their chemical synthesis counterparts, has been developed (Fernandes et al. 2003). Microbial fermentation has been wildly used to accumulate some important steroids intermediates, such as 4-androstene-3,17-dione (AD), androst-1,4-diene-3,17-dione (ADD) and 9α-hydroxy-4-AD (9OHAD) (Zhang et al. 2013; Shao et al. 2015a; Yuan et al. 2015). The 3-ketosteroid 9α-hydroxylase (KSH) and 3-ketosteroid-∆^1^-dehydrogenase (KSDD) are key enzymes in the process of microbial steroids degradation. KSH catalyzes the 9α-hydroxylation reaction of AD/ADD to 9OHAD/9α-hydroxy-4-ADD (9OHADD), whereas KSDD catalyzes the reaction of ∆^1^-dehydrogenation of AD/9OHAD to ADD/9OHADD. In this process, however, 9OHADD could subsequently form 3-hydroxy-9,10-secoandrost-1,3,5(10)-triene-9,17-dione (3HSA) by B-ring cleavage, spontaneously (Martin 1977; Kieslich 1985) (Fig. 1). Therefore, KSH combined with KSDD lead to the opening of the B-ring of steroid degradation.

The activity of KSH has been found in various bacterial genera, such as *Mycobacterium* (Wovcha et al. 1978; Brzostek et al. 2005; Van der Geize et al. 2007), *Nocardia* (Strijewski 1982), *Rhodococcus* (Van der Geize et al. 2002; Petrusma et al. 2009; Datcheva et al. 1989) and *Arthrobacter* (Dutta et al. 1992). Heterologous expression of *ksh* and characterization of KSH have been reported, and the conserved sequences analysis demonstrated that KSH is a Rieske monooxygenase. It belongs to class IA monooxygenase, including a terminal oxygenase (KshA) and a ferredoxin reductase (KshB) (Petrusma et al. 2009; Capyk et al. 2009; Arnell et al. 2007). It has been certified that KshA and KshB are essential for KSH activity by gene deletion studies of *kshA* and *kshB* (Andor et al. 2006). There were some reports about microbial fermentation from phytosterols to 9OHAD. However, due to low enzyme activities of steroids degradation pathway, they took long fermentation durations (about 120–144 h). For example, it has been reported that the mutant *Mycobacterium sp.* 2–4 M can be used to produce 9OHAD as a major product from sitosterol, with a 50 % molar yield (Donova et al. 2005). By using the resting *Rhodococcus* sp. cells to transform AD to 9OHAD, the substrate conversion ratio reached to about 85 % (Angelova et al. 1996). Generally, it is difficult to accumulate 9OHAD using fermentation method despite bacteria that can degrade steroids, because 9OHAD could be ∆^1^-dehydrogenated to 9OHADD and then spontaneously form 3HSA in these strains. Since the present of KSDD isoenzymes prevented the accumulation of intermediates (Van der Geize et al. 2000), deletion of all *ksdd* genes and overexpression of *kshA* resulted in accumulation of 9OHAD in *Mycobacterium neoaurum* (about 6.78–7.33 g L^−1^). However, the fermentation duration was more than 150 h (Yao et al. 2014). Thus, the strains which could accumulate 9OHAD, might be lack of KSDD or deficiency in KSDD (Seidel and Horhold 1992). Hence, double-stage fermentation method was developed to produce 9OHAD. The first step was the side-chain cleavage of sterols to form AD by one strain, and then the second step was 9α-hydroxylation of AD accomplished by another strain (Seidel and Horhold 1992).

Our laboratory has been devoted to using microorganisms to produce steroids intermediates with non-pollution and non-toxic biological technology (Shao et al. 2015a, 2016a). For example, we have co-expressed human 17β-hydroxysteroid dehydrogenase type 3 (17β-HSD3) and *Saccharomyces cerevisiae* glucose 6-phosphate dehydrogenase (G6PDH) to construct the NADPH regeneration system for efficient testosterone (TS) production form AD (Shao et al. 2016b). The *M. neoaurum* JC-12 (CCTCC No. M208135), capable of producing AD and ADD from phytosterol or cholesterol by fermentation method, was isolated with phytosterol as the sole carbon source from soil (Zhang et al. 2013). Genes of steroids degradation pathway from *M. neoaurum* JC-12 had been heterologous over-expressed to construct bioconversion system for steroids intermediates production. For example, cholesterol oxidase gene (*choM*) had been over-expressed in *Bacillus subtilis* for bioconversion of cholesterol to 4-cholesten-3-one (Shao et al. 2015b). 3-ketosteroid-∆^1^-dehydrogenase (*ksdd*) gene had been over-expressed in *B. subtilis* for bioconversion of AD to ADD (Zhang et al. 2013). The previous work indicated that *B. subtilis* might be a preferred host for bioconversion of steroids intermediates as compared with *M. neoaurum* strains. Hence, in this study, we cloned *kshA* and *kshB* gene from *M. neoaurum* JC-12 and first heterologously co-expressed them in *B. subtilis* 168. For efficiently bioconversion of AD to 9OHAD, glucose 1-dehydrogenase (GDH, EC 1.1.1.47, encoded by *gdh* gene) was co-expressed with KSH to construct a NADH regeneration system (Additional file 1: Fig. S1). The intracellular NADH concentration and the whole-cell bioconversion capability of recombinant *B. subtilis* were detected. This work provided a new reference for 9OHAD production.

## Methods

### Bacterial strains, plasmids and culture conditions

*Mycobacterium neoaurum* JC-12 (AD and ADD producing strain) was stored in our laboratory. *B. subtilis* 168 was purchased from Bacillus Genetic Stock Center (BGSC). The *E. Coli* and *B. Subtilis* shuttle vector pMA5 (*Hpa*II, *ColE1*, *repB*, Amp^R^, Km^R^) was preserved in our lab (Zhang et al. 2013b). *B. subtilis* strains were cultivated at 37 °C and 160 rpm in LB medium (Luria–Bertani broth) with 0.5 % (w/v, weight/volume) glucose. Kanamycin (50 mg L^−1^) was added to the growth medium for selecting the recombinants. *M. neorarum* JC-12 was grown at 30 °C and 160 rpm in liquid medium containing 0.5 % (w/v) glucose, 0.5 % (w/v) tryptone, 0.3 % (w/v) beef extract, 1.5 % (w/v) glycerol and 1.5 % (w/v) NaCl. 2 % (w/v) agar was added during cultivation on solid medium.

### Gene cloning and sequencing

Restriction enzymes and T4 DNA ligase were purchased from TaKaRa Co. (Dalian, China). Extraction and purification of plasmids were carried out by Mini Plasmid Rapid Isolation Kit (Sangon Biotech Co., Ltd., Shanghai, China). Isolation of DNA restriction fragment from agarose gels was done using the Mini DNA Rapid Purification Kit (Sangon Biotech Co., Ltd., Shanghai, China). Nucleotide sequence of *kshA* and *kshB* were analyzed by Sangon Biotech Co., Ltd. Shanghai, China. Protein and nucleotide sequences alignment were performed using the function of the BLAST server at NCBI (http://blast.ncbi.nlm.nih.gov/Blast.cgi).

### Construction of recombinant *B. subtilis* 168/pMA5-*ksh* and *B. subtilis* 168/pMA5-*ksh*–*gdh*

Primers used in this work are listed in Table 2. The construction steps of these plasmids are shown in Additional file 1: Fig. S2. The *kshA* was cloned from chromosomal DNA of *M. neoaurum* JC-12 with the forward primer KshA-F and reverse primer KshA-R. Primers were originally designed with *Nde*I and *Mlu*I restriction sites to clone *kshA* into the plasmid pMA5. Gene cloning of *kshB* was performed with forward primer KshB-F and reverse primer KshB-R1. The PCR product was ligated onto pMA5 after digested by *Bam*HI. The identification of pMA5-*kshB* was performed by digestion of *Mlu*I. Then, we got *Hpa*II-*kshB* from pMA5-*kshB* with the forward primer *Hpa*II-F1 and reverse primer KshB-R2 (containing His-Tag). The construction of pMA5-*ksh* was performed by ligation of *Hpa*II-*kshB*, digested by *Eco*RI and *Sma*I, onto pMA5-*kshA*. For co-expression of *ksh* and *gdh* to construct a NADH regeneration system, plasmid of pMA5-*gdh* (previously constructed by us, homologous over-expression of GDH from *B. subtilis* 168) was used as the template and primer pair *Hpa*II-F2/Gdh-R was used to amplify *Hpa*II-*gdh*. The construction of *B. subtilis*/pMA5-*ksh*–*gdh* was performed by ligation of *Hpa*II-*gdh* onto pMA5-*ksh*, which were digested by *Kpn*I and *Hin*dIII. The primer sequences were listed in Table 1. Transformation of *B. subtilis* cells were carried out according to the procedure described by Anagnostopoulos and Spizizen (1961). The recombinant *B. subtilis* with pMA5-*ksh* was selected by resistance to kanamycin and confirmed by DNA sequencing.

**Table 1:** Primers used in this work

Primers	Sequences (5′–3′)	Restriction sites
KshA-F	ATC**CATATG**ACTACCGAGACAGCCG	*Nde*I
KshA-R	CG**ACGCGT**TTAGTGGTGGTGGTGGTGGTGGCTCGGCTGCGCGGAC	*Mlu*I
KshB-F	CGC**GGATCC**ATGACGGAGGAGCCGCTC	*Bam*HI
KshB-R1	CGC**GGATCC**CTATTCGTCGTAGGTGACTTC	*Bam*HI
*Hpa*II-F1	ACCG**GAATTC**ATGACGGAGGAGCCGCTC	*Eco*RI
KshB-R2	CCC**CCCGGG**TTAGTGGTGGTGGTGGTGGTGTTCGTCGTAGGTGACTTC	*Sma*I
*Hpa*II-F2	ACCG**GGTACC**ATGACGGAGGAGCCGCTC	*Kpn*I
Gdh-R	ACCG**AAGCTT**TTAACCGCGGCCTGCCTGGAATGAAG	*Hind*III

The restriction enzyme sites were bold type

### Co-expression of KshA and KshB in *B. subtilis* 168 and protein purification

The recombinant plasmid pMA5-*ksh* was introduced into *B. subtilis* 168. Transformants were obtained after growing overnight on selective LB medium supplement with 50 mg L^−1^ of kanamycin. The recombinant cells were grown in LB medium (50 mL) for 24 h at 37 °C. Cell pellets (8000 rpm; 10 min; 4 °C) were washed with 100 mL of 50 mM Tris–HCl buffer (pH 7.0). Cell pellets were then resuspended in Tris–HCl buffer added with 5 mg lysozyme for 30 min and then sonicated for 10 min at 4 °C. Cell extracts were centrifuged for 30 min at 10,000 rpm in an SIGAMA 3K-15 centrifuge to remove cell debris. The purification of KSH-His was carried out by procedure described previously (Zhang et al. 2014). The final samples were verified via SDS-PAGE (12 % acrylamide).

### Enzyme activity assay

The KSH activity was detected according to the procedure described previously (Petrusma et al. 2009). For KSH enzyme activity assay, the reaction mixture consists of 50 mmol L^−1^ Tris–HCl buffer (pH 7.0), 105 µmol L^−1^ NADH, 250 µmol L^−1^ AD dissolved in 2 % methanol, and 20–25 µg co-expressed KSH (KshA and KshB). For KshB enzyme activity assay, the reaction mixture consists of 50 mmol L^−1^ Tris–HCl buffer (pH 7.0), 0.1 mmol L^−1^ 2,6-dichlorophenolindophenol, 0.25 mmol L^−1^ NADH, and 1–2 µg KshB was added to the assay. Assays were performed at room temperature. GDH activity was detected as the procedure described before (Hilt et al. 1991).

### Determination of NADH and NAD^+^ concentrations

The intracellular NADH and NAD^+^ concentrations of recombinant *B. subtilis* strains were determined according to the manufacturers’ instructions of Amplite Fluorimetric NAD/NADH Ratio Assay Kit (15263) (Sunnyvale, USA). Sample preparation was followed by the procedure described previously (Bao et al. 2015).

### Bioconversion of AD by recombinant *B. subtilis* 168

The bioconversion of AD was performed in shake flasks with the recombinant *B. subtilis* 168. The growth condition of *B. subtilis* strains was as previously described. After growing until late exponential phase (OD_600_ = 4–6), cells were collected by an SIGMA 3 K-15 centrifuge. Cell pellets were washed with 200 mL of 50 mmol L^−1^ Tris–HCl buffer (pH 7.0) for twice and then resuspended in 20 mL Tris–HCl buffer. AD and 0.2 % Tween-80 were then added into the bioconversion system. When using the NAD regeneration system as whole-cell biocatalyst, 1.5 % glucose was added as the substrate of GDH. Steroids extracted from the bioconversion solution (1 mL) by ethyl acetate were used for high-performance liquid chromatography (HPLC). For HPLC analysis, samples were diluted 5 times with ethyl acetate and filtered. Steroids were analyzed by HPLC with a reversed phase Diamonsil C18 at 35 °C using methanol–water (80: 20, v/v) solvent as mobile phase with a flow rate of 1 mL min^−1^, and subsequent detected via determination of UV absorption at 254 nm.

## Results and discussion

### *kshA* and *kshB* clone and sequence analysis

The amplification and sequences alignment of *kshA* and *kshB* genes were conducted as described in Materials and methods. The *kshA* gene of *M. neoaurum* JC-12 encodes a protein of 395 amino acids. Protein sequence analysis showed that typical conserved sequences of class IA terminal oxygenase (Van der Geize et al. 2002; Petrusma et al. 2009), the Rieske Fe_2_S_2_ binding domain (CXHX_16_CX_2_H, residues 65–87) and the non-heme Fe^2+^ motif (DX_3_DX_2_HX_4_H, residues 172–186) were found in this KshA protein. The *kshB* gene encodes 351 amino acids of KshB protein, which contains typical class IA monooxygenase reductase domains, a flavin-binding domain (RXYSL, residues 65–69), an NAD-binding domain (GSGITP, residues 129–134), and a [Fe_2_S_2_Cys_4_] domain (CX_4_CX_2_CX_29_C, residues 288–336). The sequences of *kshA* (GenBank: KR611532.1) and *kshB* (GenBank: KR611533.1) genes were then submitted to the GenBank database.

### Co-expression, purification and characterization of KshA and KshB

The over-expressed KSH from *M. neoaurum* JC-12 in *E. coli* with the vector pET28a had been executed in our previous study, the recombinant proteins mainly existed in the form of inclusion bodies and no KSH activity was detected. Thus, the recombinant plasmids of pMA5-*kshA*, pMA5-*kshB* and pMA5-*ksh* (i.e., pMA5-*kshA*–*kshB*), which allowed the gene *kshA* and *kshB* to be expressed under the control of *Hpa*II promoter, were constructed. After transformation of the recombinant plasmids into *B. subtilis* 168, they were then selected by using kanamycin as the selectable marker.

**Fig. 2:**
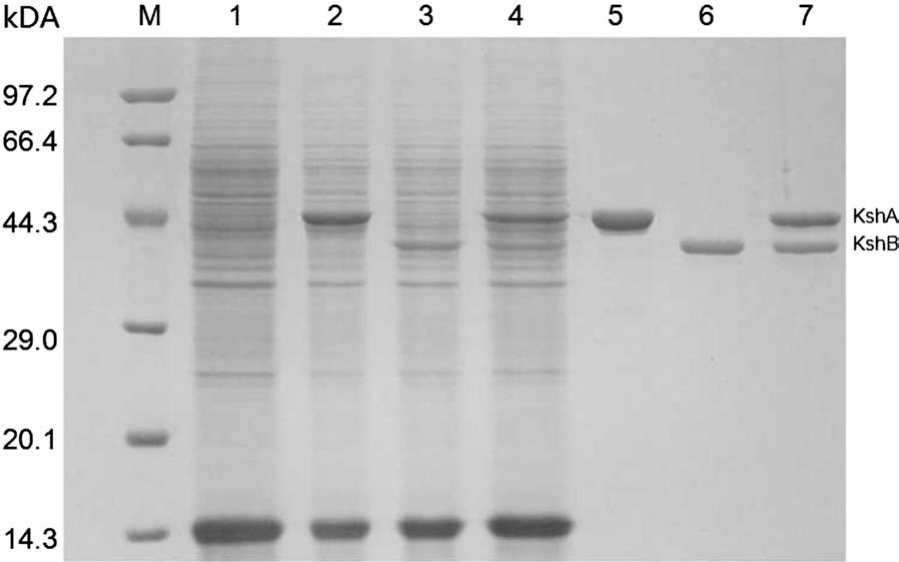
SDS-PAGE analysis of cell-free extract and purified KshA and KshB. Lanes: *M* protein marker (Takara Biotechnology Co., Ltd., Dalian, China); *1* cell-free extract of *B. subtilis* 168; *2* cell-free extract of *B. subtilis* 168/pMA5-*kshA*; *3* cell-free extract of *B. subtilis* 168/pMA5-*kshB*; *4* cell-free extract of *B. subtilis* 168/pMA5-*ksh*; *5* purified KshA (45.1 kDa); *6* purified KshB (37.8 kDa); *7* copurified KshA and KshB

In this study, the possible expression of KSH by recombinant *B. subtilis* was investigated by SDS-PAGE (Fig. 2), and the analysis of proteins showed that KshA and KshB were successfully expressed. The enzyme activity of KshB, the reductase component of KSH, accepts electrons from NAD(P)H and transfers it to oxygenase component KshA, had been successfully detected in *B. subtilis* 168/pMA5-*kshB* with *B. subtilis* 168 as control (data not shown). The previous study strongly suggested that the cooperation of KshA and KshB are critically important for maintaining KSH activity (Petrusma et al. 2009). Hence, KSH activity could be only detected when KshA and KshB were co-expressed (Table 2). The KSH activity of *B. subtilis* 168/pMA5-*ksh* was 0.57 U (mg total protein)^−1^. However, KSH activity of *M. neoaurum* JC-12 were only about 0.02 U (mg total protein)^−1^, suggesting co-expression of KshA and KshB in engineered *B. subtilis* successfully improved KSH activity.

**Table 2:** Enzyme activity assay for KSH and GDH

Strains	KSH activity U (mg total protein)^−1^	GDH activity U (mg total protein)^−1^
*M. neoaurum* JC-12	0.02 ± 0.01	–
*B. subtilis* 168	NT	0.03 ± 0.01
*B. subtilis* 168/pMA5-*kshA*	NT	0.03 ± 0.01
*B. subtilis* 168/pMA5-*ksh*	0.57 ± 0.02	0.03 ± 0.01
*B. subtilis* 168/pMA5-*ksh*–*gdh*	0.53 ± 0.02	0.35 ± 0.02
Purified KshA	NT	–
Co-purified KshA and KshB	2.41 ± 0.05	–

All assays were performed in triplicate

NT, enzyme activity was not detectable; –, not detect

KshA and KshB were purified with the C-terminal His-tag and showed KSH activity of 2.41 U mg^−1^. The maximum KSH activity of co-purified KshA and KshB was observed at 33 °C and pH 7.0. Measurements showed that a rather narrow pH range was needed for KSH activity. However, the enzyme was not stable when stored at −20 and 0 °C, and the KSH activity was reduced by 28 and 67 % after 24 h, respectively. No metal ions were found enhanced the KSH activity conspicuously. On the contrary, however, most metal ions were inhibitors of KSH, such as Fe^3+^, Co^2+^, Zn^2+^, Cu^2+^, Zn^2+^ and Ni^2+^, in which Zn^2+^ could inhibit KSH activity completely.

### Construction of NADH regeneration system for biocatalysis of AD to 9OHAD

**Fig. 3:**
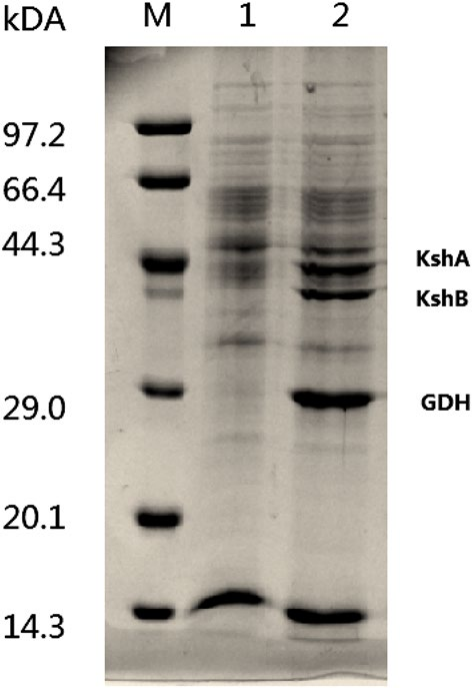
SDS-PAGE analysis of KSH and GDH co-expressed in *B. subtilis*. Lanes: *M* protein marker (Takara Biotechnology Co., Ltd., Dalian, China); *1* cell-free extract of *B. subtilis* 168; *2* cell-free extract of *B. subtilis* 168/pMA5-*ksh*–*gdh*

GDH from *B. subtilis* 168 and KSH from *M. neoaurum* JC-12 were co-expressed to construct the NADH regeneration system (*B. subtilis* 168/pMA5-*ksh*–*gdh*). SDS-PAGE analysis proved GDH and KSH were successfully co-expressed in *B. subtilis* (Fig. 3). Compared to *B. subtilis* 168, of which GDH activity was 0.03 U (mg total protein)^−1^, the KSH and GDH activities of *B. subtilis* 168/pMA5-*ksh*–*gdh* were 0.53 and 0.35 U (mg total protein)^−1^, respectively, indicating the functional over-expression of GDH.

**Fig. 4:**
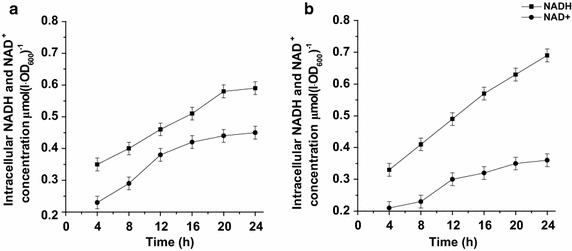
The intracellular NADH and NAD^+^ concentrations of recombinant strains *B. subtilis* 168/pMA5-*ksh* (**a**) and *B. subtilis* 168/pMA5-*ksh*–*gdh* (**b**) (all assays were performed by three independent biological repeats, and the standard deviations of the biological replicates were represented by *error bars*)

The significant role of cofactors in the biocatalysts was proved by comparing the intracellular concentrations of NAD^+^ and NADH in the recombinant *B. subtilis* strains (Fig. 4). The results showed that the intracellular NADH pool in recombinant *B. subtilis* 168/pMA5-*ksh*–*gdh* was improved (17 %) as compared with *B. subtilis* 168/pMA5-*ksh* by over-expression of GDH, suggesting the NADH regeneration system was successfully constructed.

### Steroid transformation analysis of the recombinant *B. subtilis* 168/pMA5-*ksh* and *B. subtilis* 168/pMA5-*ksh*–*gdh*

The biosynthesized 9OHAD in *M. neoaurum* strains could be subsequently transformed to 9OHADD, which then undergoes a nonenzymatic ring cleavage to form 3HSA. However, the recombinant *B. subtilis* 168 could catalyze the transformation of 9OHAD from AD in one step without any degradation of 9OHAD. Moreover, *B. subtilis* has been wildly used and regarded as a safe strain in industries (Schallmey et al. 2004).

**Fig. 5:**
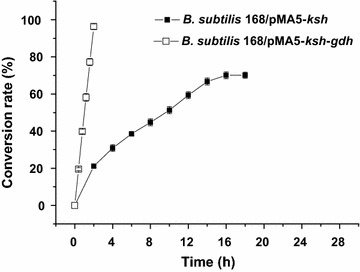
Conversion rate of AD to 9ODAD by recombinant strains *B. subtilis* 168/pMA5-*ksh* (X1) and *B. subtilis* 168/pMA5-*ksh*–*gdh* (X2) (all assays were performed by three independent biological experiments, and the standard deviations of the biological replicates were represented by *error bars*)

While using whole-cells as biocatalyst, 1 g L^−1^ AD was added as substrate to validate the bioconversion efficiency of different strains. The results showed that *B. subtilis* 168/pMA5-*ksh* and *B. subtilis* 168/pMA5-*ksh*–*gdh* successfully catalyzed the transformation of AD to 9OHAD (Fig. 5). However, as controls, no 9OHAD was detected during AD bioconversion by whole-cells of *M. neoaurum* JC-12, *B. subtilis* 168, *B. subtilis* 168/pMA5-*kshA* and *B. subtilis* 168/pMA5-*kshB*. Although both *B. subtilis* 168/pMA5-*ksh* and *B. subtilis* 168/pMA5-*ksh*–*gdh* showed significant improved KSH activity, NAD^+^ was only continuously regenerated by GDH in *B. subtilis* 168/pMA5-*ksh*–*gdh* to keep a persistently 9OHAD productivity. The maximum conversion rate (g g^−1^) of *B. subtilis* 168/pMA5-*ksh* was 70.1 % at 16 h, while *B. subtilis* 168/pMA5-*ksh*–*gdh* showed a conversion rate (g g^−1^) of 96.3 % at 2 h, indicating cofactor regeneration increased the conversion rate of the biocatalyst. After the batch conversion of *B. subtilis* 168/pMA5-*ksh*–*gdh*, the pH of the reaction mixture was decreased from 7.0 to about 6.3. Thus, the pH variation had little effect on this system.

**Fig. 6:**
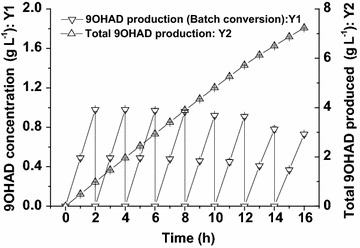
Repeated batch conversion of AD to 9ODAD by recombinant strain *B. subtilis* 168/pMA5-*ksh*–*gdh.* Y1: 9ODAD production of batch conversion, Y2: total 9ODAD production of repeated conversion (all assays were performed by three independent biological experiments, and the standard deviations of the biological replicates were represented by *error bars*)

From above experiments, *B. subtilis* 168/pMA5-*ksh*–*gdh* was expected a good candidate for transforming of AD to 9OHAD. However, due to the low solubility of the steroid substrates and products in aqueous conversion system, the repeated batch strategy of bioconversion from AD to 9OHAD was done in this work. While using 1 g L^−1^ AD as substrate for repeated batch bioconversion, the whole-cells of *B. subtilis* 168/pMA5-*ksh*–*gdh* could continuously transform total 8 g L^−1^ AD to about 7.23 g L^−1^ 9OHAD within 16 h with a conversion rate (g g^−1^) of 90.4 % and productivity of 0.45 g (L h)^−1^ (Fig. 6). However, the enzyme activity of whole-cell biocatalyst decreased greatly (left about 38.9 % KSH activity and 30.6 % GDH activity) after 16 h. Thus the conversion duration was controlled within 16 h. In summary, the successful expression of *M. neoaurum* KSH in *B. subtilis* and the construction of NADH regeneration system made it possible to achieve one-step efficient transformation of AD to 9OHAD.

## Conclusions

9OHAD, an important precursor in the synthesis of steroid pharmaceuticals, can be produced by microorganisms using fermentation method. However, due to the long fermentation duration and low substrate conversion rate, the productivity of biosynthesis of 9OHAD cannot meet the need of industrial production. This work cloned and over-expressed *M. neoaurum* KSH and *B. subtilis* GDH to construct a NADH regeneration system (*B. subtilis* pMA5-*ksh*–*gdh*), which could efficiently transform AD to 9OHAD. By using the NADH regeneration system as a biocatalyst integrated the repeated batch conversion strategy, 9OHAD production was improved to 7.23 g L^−1^ with a conversion rate of 90.4 % and productivity of 0.45 g (L h)^−1^. The results demonstrated that the NADH regeneration system of recombinant *B. subtilis* strain can be used in 9OHAD production. However, the low solubility of the steroid substrates in aqueous conversion system limits extremely the bioconversion rate. Thus, aqueous-organic two-phase systems and cloud point systems will be considered to be applied in the future research to improve the conversion rate of AD to 9OHAD (Wang et al. 2005).

## Additional file

10.1186/s40064-016-2871-4 The NADH regeneration system constructed by this work. **Figure S2.** Construction steps of the plasmids used in this work.
