# Supplementary material for: Efficient 9α-hydroxy-4-androstene-3,17-dione production by engineered Bacillus subtilis co-expressing Mycobacterium neoaurum 3-ketosteroid 9α-hydroxylase and B. subtilis glucose 1-dehydrogenase with NADH regeneration
Source: Springerplus. 2016 Jul 29;5(1):1207. doi: 10.1186/s40064-016-2871-4 (PMC4967059; doi:10.1186/s40064-016-2871-4)
Supplement: Supplementary file 1 — 10.1186/s40064-016-2871-4 The NADH regeneration system constructed by this work. Figure S2. Construction steps of the plasmids used in this work. [file 40064_2016_2871_MOESM1_ESM.docx]

**Efficient** **9α-hydroxy-4-androstene-3,17-dione production by engineered *Bacillus subtilis* co-expressing *Mycobacterium neoaurum* 3-ketosteroid 9α-Hydroxylase and *B. subtilis* glucose 1-dehydrogenase with NADH regeneration**

Xian Zhang^a^, Zhiming Rao^a*^, Lele Zhang^b^, Meijuan Xu^a^, Taowei Yang^a^

*a. The Key Laboratory of Industrial Biotechnology of Ministry of Education, School of Biotechnology,* *Jiangnan University, Wuxi, Jiangsu 214122, P. R. China*

*b.* *Jiangnan University (Rugao) Food Biotechnology Research Institute, Jiangsu Industrial Technology Research Institute, Rugao, Jiangsu 226500, P. R. China*

*** Correspondence

Zhiming Rao

Tel: +86-510-85916881

E-mail: raozhm@jiangnan.edu.cn

**Additional file**

**Figure S1** The NADH regeneration system constructed by this work.

**Figure S2** Construction steps of the plasmids used in this work.
